# SCR‐7952, a highly selective MAT2A inhibitor, demonstrates synergistic antitumor activities in combination with the *S*‐adenosylmethionine‐competitive or the methylthioadenosine‐cooperative protein arginine methyltransferase 5 inhibitors in methylthioadenosine phosphorylase‐deleted tumors

**DOI:** 10.1002/mco2.705

**Published:** 2024-09-20

**Authors:** Zhiyong Yu, Yi Kuang, Liting Xue, Xuan Ma, Tingting Li, Linlin Yuan, Mengying Li, Grace Xue, Zhen Li, Feng Tang, Jianxing Tang, Jinwen Shan, Weijie Wang, Renhong Tang, Feng Zhou

**Affiliations:** ^1^ State Key Laboratory of Neurology and Oncology Drug Development Nanjing China; ^2^ Department of Preclinical Research Simcere Zaiming Pharmaceutical Co., Ltd. Shanghai China; ^3^ Department of Thoracic Surgery The Affiliated Xiangshan Hospital of Wenzhou Medical University Wenzhou Zhejiang China; ^4^ Weston High School Weston Massachusetts USA

**Keywords:** drug combination, MAT2A inhibitor, MTAP‐deleted cancer, PRMT5

## Abstract

The metabolic enzyme methionine adenosyltransferase 2A (MAT2A) was found to elicit synthetic lethality in methylthioadenosine phosphorylase (*MTAP*)‐deleted cancers, which occur in about 15% of all cancers. Here, we described a novel MAT2A inhibitor, SCR‐7952 with potent and selective antitumor effects on *MTAP*‐deleted cancers in both in vitro and in vivo. The cryo‐EM data indicated the high binding affinity and the allosteric binding site of SCR‐7952 on MAT2A. Different from AG‐270, SCR‐7952 exhibited little influence on metabolic enzymes and did not increase the plasma levels of bilirubin. A systematic evaluation of combination between SCR‐7952 and different types of protein arginine methyltransferase 5 (PRMT5) inhibitors indicated remarkable synergistic interactions between SCR‐7952 and the *S*‐adenosylmethionine‐competitive or the methylthioadenosine‐cooperative PRMT5 inhibitors, but not substrate‐competitive ones. The mechanism was via the aggravated inhibition of PRMT5 and FANCA splicing perturbations. These results indicated that SCR‐7952 could be a potential therapeutic candidate for the treatment of *MTAP*‐deleted cancers, both monotherapy and in combination with PRMT5 inhibitors.

## Graphical abstract

We discovered a potent and specific allosteric MAT2A inhibitor SCR‐7952. SCR‐7952 selectively inhibited the growth of *MTAP*‐deleted cancers, via the regulation of PRMT5 activity. The cryo‐EM indicated an allosteric binding site. Furthermore, the combination of SCR‐7952 with SAM‐competitive or MTA‐cooperative PRMT5 inhibitors supported further investigation and potential development of an effective strategy for larger therapeutic windows on *MTAP*‐deleted cancers.

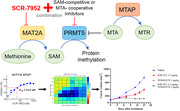

## 1 INTRODUCTION

The methionine cycle is a crucial process for many cellular functions, including polyamine synthesis, DNA synthesis, redox balance, and histone methylation.^1^ Cancer cells are highly dependent on the methionine cycle, and methionine depletion or pharmacological inhibition of methionine cycle enzymes is sufficient to result in long‐term loss of tumorigenic potential.^2^ Methionine adenosyltransferase 2A (MAT2A), one of the rate‐limiting enzymes, catalyzes methionine into *S*‐adenosylmethionine (SAM), which is the global methyl‐donor for transmethylation and biosynthesis reactions.^3^, ^4^ The increase of MAT2A was observed in several malignancies, including lung, colon, liver, gastric, and leukemic cancers, which provides advantages for mitogens and cell growth.^2^, ^5^, ^6^, ^7^, ^8^

MAT2A was found as a synthetic lethal target in methylthioadenosine phosphorylase (*MTAP*)‐deficient cancer cells, and the mechanism involves SAM‐utilizing protein arginine methyltransferase 5 (PRMT5). MTAP is the only metabolic enzyme in the methionine salvage pathway for the degradation of methylthioadenosine (MTA).^9^ The deletion of MTAP leads to an accumulation of MTA, which acts as a natural inhibitor of PRMT5. PRMT5 is a critical methyltransferase that utilizes the methyl donor SAM for symmetric dimethylarginine (SDMA) posttranslational modifications, and participates in a series of essential cellular functions including transcription, splicing, RNA biology, damage response, and cell metabolism.^10^ It is hypothesized that a reduction of intracellular SAM due to the inhibition of MAT2A in *MTAP*‐deficient cells leads to sensitization of PRMT5 to further inhibition, thus impairing PRMT5 downstream signals. Silencing or inhibition of MAT2A results in cell death selectively in *MTAP*‐deleted cancers via the block of SAM biosynthesis, but exhibits limited influence on normal cells with a reduced concentration of intracellular MTA.^11^, ^12^ Since *MTAP*‐deletion occurs at high frequency in almost 15% of all human cancer types,^12^ the synthetic lethality emerges MAT2A as a potential target for cancer treatment.

Currently, several substrate‐competitive or allosteric MAT2A inhibitors have been discovered, such as methionine analogs, arylquinazolinone derivatives, and pyrazolopyrimidinone derivatives.^13^, ^14^ And some literatures reported a series of pyrrolopyridone MAT2A inhibitors by structure‐based drug discovery.^15^, ^16^ However, the in vivo applications were mostly limited by moderate potency, low MAT2A specificity, or poor pharmacokinetic properties. The first druglike MAT2A allosteric inhibitor, PF‐9366 showed a potent inhibition on MAT2A (IC_50_ of 420 nM in a chemical assay), but an inadequate antiproliferative effect on Huh‐7 cells (IC_50_ of 10 µM), presumably due to the upregulation of MAT2A expression as a feedback mechanism after PF‐9366 treatment.^17^ A more potent MAT2A inhibitor, AG‐270 is under early clinical evaluation (NCT03435250, clinical trials.gov). It effectively inhibited MAT2A and suppressed the growth of *MTAP*‐null tumors in the preclinical study.^14^ The improved potency probably enabled AG‐270 to maintain antiproliferative effects despite cellular adaptation. Still, due to the off‐targeted activity on UGT1A1 (IC_50_ of 1.1 µM), AG‐270 led to hyperbilirubinemia and may limit the dose escalation.^18^ Therefore, developing the new MAT2A inhibitors with high potency and better selectivity is still needed.

Several types of PRMT5 inhibitors have been discovered, including SAM‐competitive, MTA‐cooperative, and substrate‐competitive ones. But clinical studies reported that PRMT5 inhibitors commonly cause pancytopenia, thrombocytopenia neutropenia as dose‐limiting toxicity.^19^ The findings that PRMT5 is also a synthetic lethal vulnerability in *MTAP*‐deficient tumors indicates a combination strategy of MAT2A inhibitors and PRMT5 inhibitors. The mechanism indicated high safety in normal cells with MTAP expression. However, just a few publications reported the dual inhibition of MAT2A and PRMT5.^20^, ^21^ The researches only investigated the combination with specific MTA‐cooperative PRMT5 inhibitors, and lacked of systematic evaluations and further mechanism.

Herein, we discovered a highly selective MAT2A inhibitor SCR‐7952. SCR‐7952 substrate‐noncompetitively inhibits MAT2A activity both in the biochemical assay and in cells. SCR‐7952 selectively suppressed *MTAP*‐deleted cancer cell proliferation in vitro and tumor growth in vivo, via regulation of PRMT5 activity and its downstream splicing perturbations. The combination strategy with PRMT5 inhibitors was evaluated, and different types of PRMT5 inhibitors exhibited different synergistic effects with SCR‐7952. Remarkable synergistic interactions were observed between SCR‐7952 and the SAM‐competitive or the MTA‐cooperative PRMT5 inhibitor, probably via the aggravated inhibition of PRMT5.

## 2 RESULTS

### 2.1 Identification of SCR‐7952 as a potent MAT2A inhibitor

**FIGURE 1:**
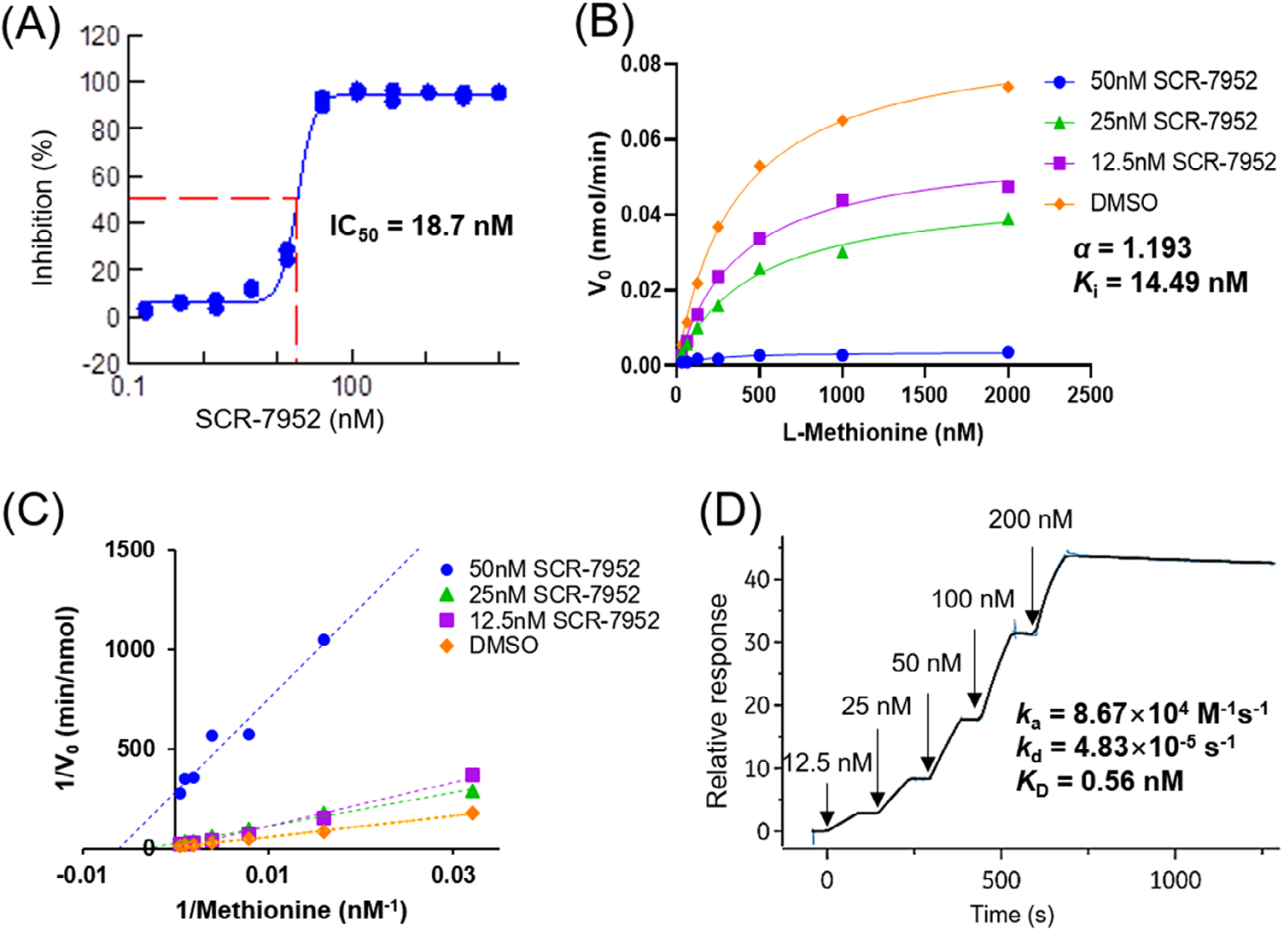
SCR‐7952 inhibition on MAT2A. (A) Inhibition of MAT2A enzyme in biochemical assay, (B) and (C) kinetic analysis of SCR‐7952 on MAT2A, and (D) binding response of SCR‐7952 to MAT2A protein by SPR.

Through a high‐throughput screening assay, a potent MAT2A inhibitor SCR‐7952 (7‐chloro‐5‐(2‐cyclopropylpyridin‐3‐yl)‐1‐methyl‐1,5‐dihydro‐4H‐imidazo[4,5‐c]quinolin‐4‐one) was identified from a series of small molecules. SCR‐7952 dose‐dependently inhibited the MAT2A enzymatic activity with a high potency, which was 3.7‐fold more potent than the previously reported MAT2A inhibitor AG‐270 (IC_50_ 18.7 vs. 68.3 nM) (Figure 1A and Table S2). The kinetic mechanism of SCR‐7592 inhibition in the presence of substrate methionine was evaluated using the Michaelis–Menten approach and the *K*_i_ value was 14.49 nM (Figure 1B). The α value of 1.193 indicated a noncompetitive inhibition effect of SCR‐7952 for methionine, which was further demonstrated from the unvarying *K*_m_ in the Lineweaver–Burk plot (Figure 1C).

The interaction between SCR‐7952 with MAT2A was then evaluated using a surface plasmon resonance (SPR) assay. The association and dissociation rate constants, *k*_a_ and *k*_d_ values were determined as 8.67 × 10^4^ M^−1^ s^−1^ and 4.83 × 10^−5^ s^−1^, respectively, using a single‐cycle kinetic model (Figure 1D). The dissociation equilibrium constant (*K*_D_) was determined as 0.56 nM, indicating a high binding affinity of SCR‐7952 to MAT2A protein.

**FIGURE 2:**
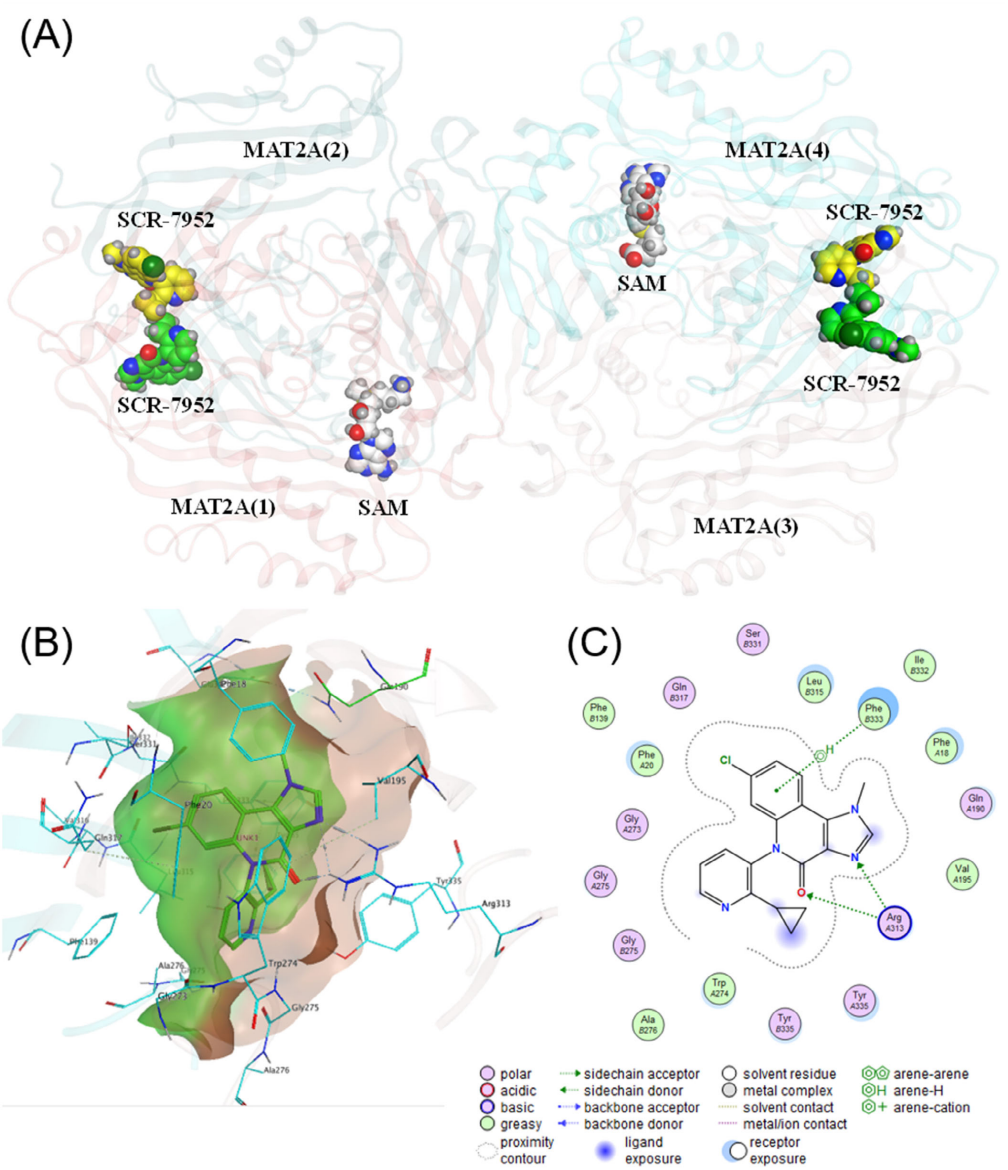
Crystal structure of MAT2A bound to SCR‐7952. (A) Cryo‐EM structure for MAT2A·SAM·SCR‐7952 (PDB ID 8XJ2), (B) the surface of the SCR‐7952 binding pocket (green for one monomer and pink for the second monomer), and (C) schematic diagram of two‐dimensional interaction for SCR‐7952 (Drawing in Molecular Operating Environment).

To identify the binding site of SCR‐7952 on MAT2A, we obtained the co‐crystal structure of MAT2A protein in complex with SCR‐7952 and SAM using cryogenic electron microscopy (cryo‐EM). The cryo‐EM data showed the ternary complex of MAT2A bound to SAM and SCR‐7952, determined at 3.16 Å resolution (Figure 2A). There were four molecules of SCR‐7952 bound at the interface of an obligate MAT2A tetramer, one molecule of SCR‐7952 per monomer of MAT2A (Figure 2B). And two molecules of substrate SAM were observed bound to a MAT2A tetramer at different binding sites from SCR‐7952. Prior crystallographic studies demonstrated that in the absence of inhibitors, two competent active sites could be observed between two MAT2A monomers (Figure S1A). However, after SCR‐7952 bound to the allosteric pocket, there was only one active binding site of SAM remained between two monomers. The α‐helix gating loop (109‐140) at the active site of MAT2A closed after inhibitor binding, and the reaction product was trapped.^22^, ^23^ The alignment of MAT2A·SAM·SCR‐7952 and MAT2A·SAM·MAT2B also showed that SCR‐7952 binds at the same allosteric pocket as MAT2B does, rather than the binding site of the substrate (Figure S1B). The cryo‐EM structure supported the noncompetitive binding of SCR‐7952 for methionine. The mechanism was similar to previously reported MAT2A inhibitors AG‐270 and PF‐9366, respectively.

SCR‐7952 possesses three parallel rings with more stable conformation and interactions with MAT2A, and maintains two hydrogen bond interactions with Arg313 on one monomer and CH‐Pi interactions with Phe333 on the second monomer (Figure 2B,C). The cyclopropyl of SCR‐7952 has good hydrophobic interactions with Tyr335 and Trp274 on one monomer. The pyridine ring of SCR‐7952 is bound in a pocket surrounded by Gly273, Gly275, and Trp274 on one monomer and Gly275 and Ala276 on the second monomer. The chlorine group of SCR‐7952 is buried in a hydrophobic pocket composed of aliphatic portions of Phe20 on one monomer and Ser331, Gln317, Phe139, Leu315, and Phe333 on the second monomer. The cryo‐EM structure confirmed critical interactions of SCR‐7952 with MAT2A.

We compared the binding pose of SCR‐7952 and AG‐270 (Figure S1C). SCR‐7952 and AG‐270 both bind to the same pocket. The structure of SCR‐7952 is smaller than AG‐270, but the activity is three‐fold more potent, suggesting that SCR‐7952 forms different and critical interactions with MAT2A. In detail, SCR‐7952 forms two hydrogen bonds with Arg313, one on nitrogen (new interaction) and the other on carbonyl (same with AG‐270). Another newly formed interaction is the CH‐Pi between SCR‐7952 and Phe333. The cyclopropyl fragment from SCR‐7952 binds to a small hydrophobic pocket composed of Phe333 and Try 335, which is not occupied by AG‐270 (Figures 2 and S1C). These newly formed interactions could be responsible for the higher binding potency of SCR‐7952 than AG‐270.

### 2.2 Characterization of cell methylation and proliferation

**FIGURE 3:**
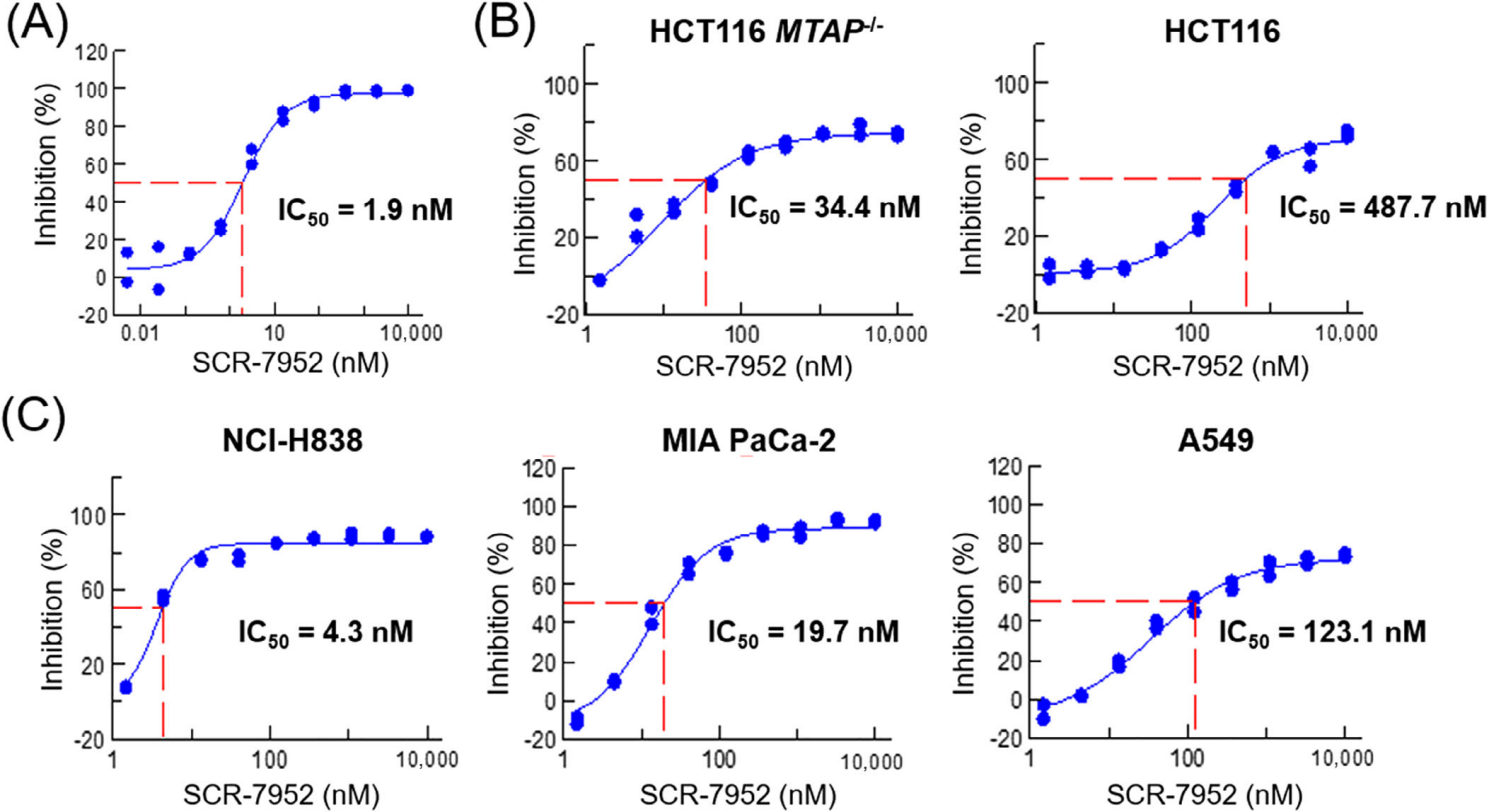
Reduction of SAM and antiproliferation of SCR‐7952 on *MTAP*‐deleted cells. (A) The reduction in cellular SAM levels after 6 h of treatment with SCR‐7952 in HCT116 *MTAP* ^−/−^ cells. (B) The 6‐day antiproliferative effects of SCR‐7952 in HCT116 *MTAP* ^−/−^ and WT cells, and (C) in *MTAP*‐deleted cell lines NCI‐H838, MIA PaCa‐2, and A549 cells.

To bridge the biochemical data to intracellular response, the impact of MAT2A was evaluated in HCT116 *MTAP^−/−^* cells, by measuring the SAM production post SCR‐7952 treatment. Consistent with the high potency in biochemistry assay, treatment of SCR‐7952 led to a dose‐dependent reduction of cellular SAM levels with IC_50_ of 1.9 nM, three‐fold more potent than AG‐270 (IC_50_ 5.8 nM) (Figure 3A).

Given the synthetic lethality of MAT2A and MTAP, the effect of SCR‐7952 on cell proliferation was evaluated in both HCT116 *MTAP^−/−^* and WT cells (Figure 3B and Table S2). SCR‐7952 exhibited a potent antiproliferation effect on HCT116 *MTAP^−/−^* cells, with IC_50_ of 34.4 nM after 6‐day treatment. As expected, SCR‐7952 demonstrated low‐to‐moderate antiproliferation activity on *MTAP‐*WT HCT116 parent cells (IC_50_ of 487.7 nM). The selective antiproliferation of *MTAP^−/−^* cells indicated a viable therapeutic approach to *MTAP* naturally deleted cancer cells. Then, we further explored the antiproliferative effects of SCR‐7952 on several *MTAP*‐deleted or WT cell lines (Figures 3C and S2). SCR‐7952 tended to inhibit the growth of *MTAP*‐deleted cell lines H838, MIA PaCa‐2, and A549, with IC_50_ of 4.3, 19.7, and 123.1 nM, respectively. However, the antiproliferation effect on *MTAP* WT cell line WI‐38 (human fetal lung fibroblast cells) was much higher, with IC_50_ of 988.5 nM, respectively. It was demonstrated that SCR‐7952 possessed *MTAP*‐null selective antiproliferative activity.

### 2.3 Regulation of PRMT5 and its downstream

**FIGURE 4:**
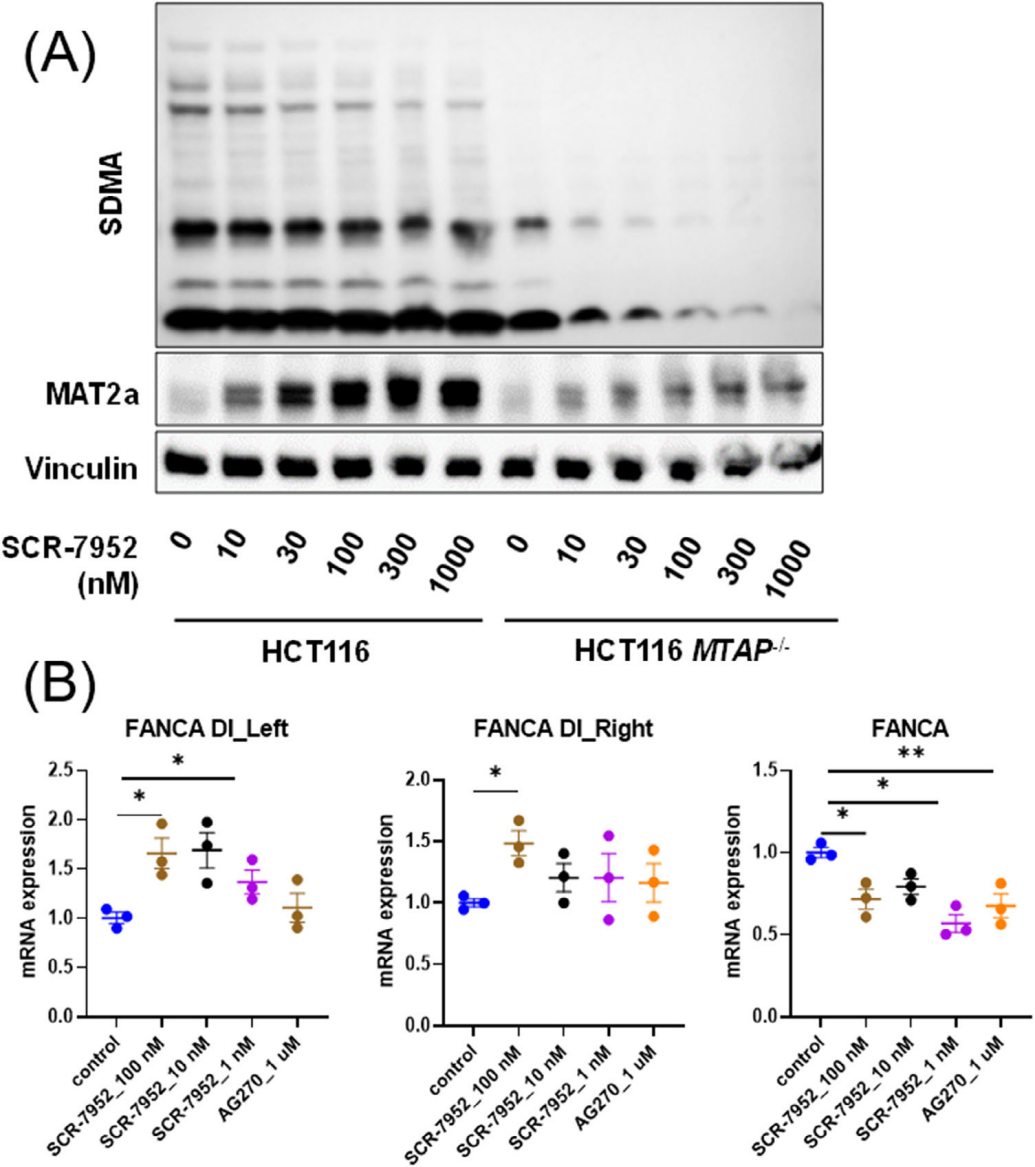
Regulation of PRMT5 downstream and splicing events. (A) Immunoblot analysis of SDMA and MAT2A after SCR‐7952 treatment for 96 h, and (B) RT‐PCR of expressions of FANCA and the DI‐containing transcripts after SCR‐7952 or AG‐270 treatment for 72 h in HCT116 WT or *MTAP*‐deleted cells. The protein grayscale analysis was performed by ImageJ 1.54d, normalized to vinculin.

To evaluate the feedback effect of SCR‐7952, the expression of MAT2A protein was assessed after SCR‐7952 treatment for 96 h. Consistent with previous MAT2A inhibitors, SCR‐7952 upregulated the expression of MAT2A protein in a dose‐dependent manner in both HCT116 *MTAP^−/−^* and WT cells (Figure 4A). However, the upregulation of MAT2A in *MTAP^−/−^* cells was much lower than that in WT cells. Although with negative feedback regulation, SCR‐7952 possessed promising inhibitory activity on cancer cell growth. It was hypothesized that the high potency of SCR‐7952 confined the feedback effect, and thus maintained its antiproliferative effect on *MTAP^−/−^* cells despite the cellular adaptation.

The methyltransferase PRMT5 utilizes SAM as the substrate for arginine methylation of regulatory proteins, functioning in epigenetic regulation of gene expression, splicing, and signal transduction.^10^ Previous studies have demonstrated that the inhibition of MAT2A impairs the activity of PRMT5, thus leading to the deregulation of RNA splicing.^12^ The PRMT5‐dependent mark SDMA was assessed in *MTAP* ^−/−^ and WT cells after 96 h treatment with SCR‐7952 (Figure 4A). Significant dose‐dependent reduction of SDMA was observed in *MTAP* ^−/−^ cells, indicating the inhibition of PRMT5 activity. However, the expression changes of SDMA in WT cells were not as significant as those in *MTAP* ^−/−^ cells.

PRMT5 inhibition is closely related to dysregulation of splicing, which leads to a large increase in splicing events, including detained introns (DIs). It was reported that MAT2A inhibition regulates Fanconi anemia (FA) DNA repair pathway by the accumulation of DI transcripts.^12^ The expressions of FANCA and the DI‐containing transcripts were assessed by RT‐PCR (Figure 4B). SCR‐7952 treatment significantly upregulated the expression of FANCA DI‐containing transcripts, and reduced total levels of FANCA mRNA. It was consistent with previous reports, indicating that MAT2A inhibitor SCR‐7952 dysregulates splicing downstream of MAT2A and triggers DNA damage through its impact on PRMT5 activity.

Further insight into selectivity was evaluated in a functional panel of 47 representative targets closely related to human life activities, including G protein‐coupled receptors, transporters, ion channels, kinases, nuclear hormone receptor targets, and nonkinase enzymes (Table S3). SCR‐7952 did not exhibit significant pharmacological effects, either agonistic or antagonistic activities on these targets, even at a very high concentration of 10 µM. These results indicated that the reduction of SCR‐7952 in SAM was an on‐target effect with low off‐target risks.

### 2.4 Antitumor effects in an *MTAP* ^−/−^ xenograft mouse model

An efficacy study in an HCT116 *MTAP* ^−/−^ subcutaneous xenograft mouse model was performed to evaluate the antitumor effect of SCR‐7952, with once daily (q.d.) oral dosing of 0.25, 0.5, 1.0, or 3.0 mg/kg, respectively.

**FIGURE 5:**
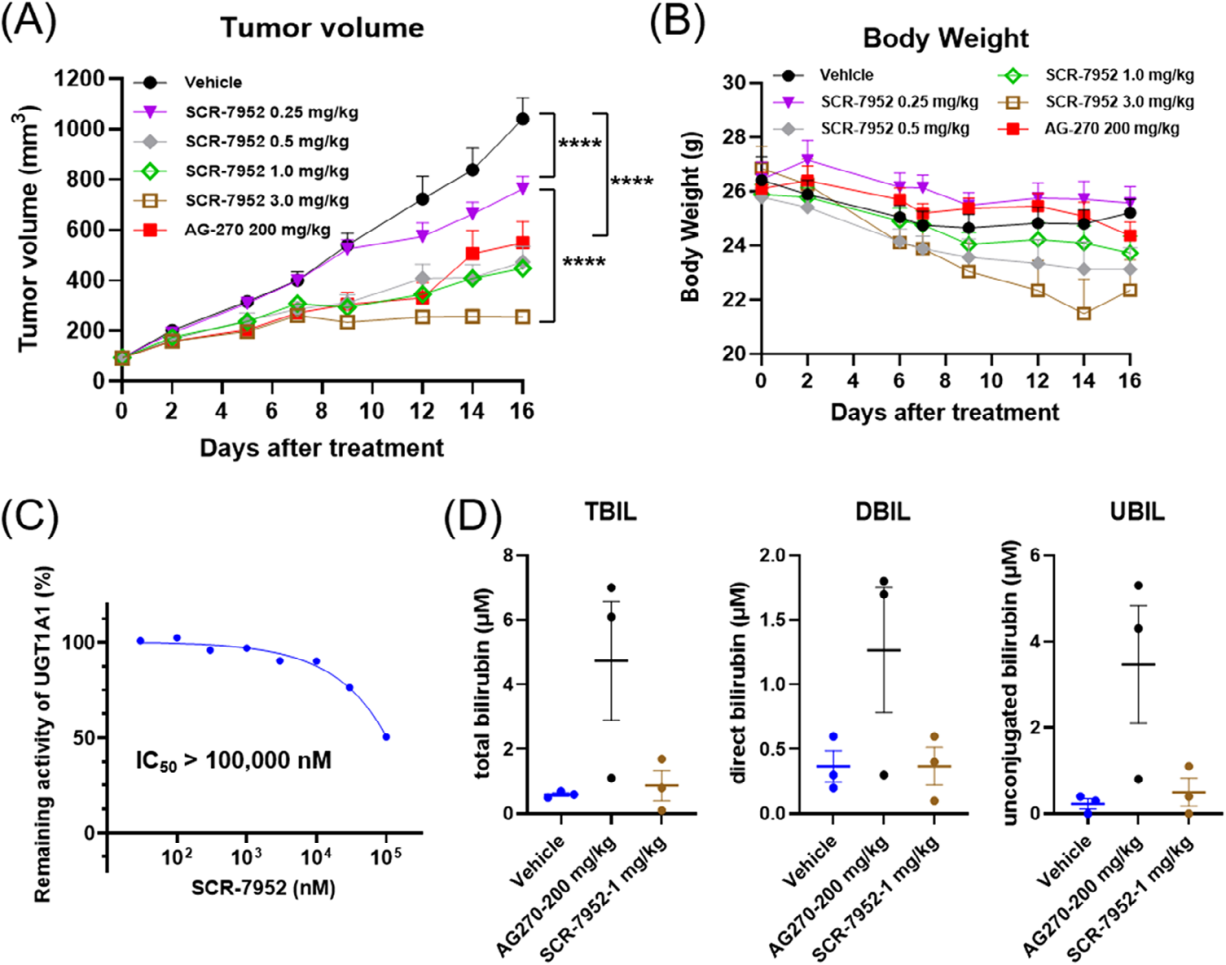
In vivo effects of SCR‐7952. (A) Tumor volumes and (B) body weights of HCT116 *MTAP* ^−/−^ tumor‐bearing mice treated with SCR‐7952, AG‐270, or vehicle (mean ± SEM, *n* = 7). (C) The effect of SCR‐7952 on human UGT1A1 in a biochemical assay. (D) The levels of plasma total bilirubin (TBIL), direct bilirubin (DBIL), and unconjugated bilirubin (UBIL) of mice 2 h after the final dose (mean ± SEM, *n* = 3). *p* Values were calculated using two‐way ANOVA. ****p < 0.0001.

Compared with the vehicle group, oral administration of SCR‐7952 led to a significant dose‐dependent reduction in tumor growth in the xenograft mouse model (Figure 5A). SCR‐7952 at 0.5 and 1 mg/kg presented similar tumor growth inhibition to AG‐270 at 200 mg/kg (TGI 60.0%, 62.7 and 52.0%, respectively). SCR‐7952 at 3.0 mg/kg presented a stronger tumor growth inhibition than that of AG‐270 at 200 mg/kg (TGI 82.9 vs. 52.0%, *p *< 0.0001). The mice were tolerable in the treatment group, and no other adverse effects or animal abnormalities were observed during the study (Figure 5B).

In a satellite group, we evaluated the SAM levels in HCT116 *MTAP* ^−/−^ xenograft tumors and mice plasma after SCR‐7952 treatment to demonstrate the in vivo target inhibition (Figure S3). The results showed a significant decrease of SAM levels both in tumors and plasma, with 53.56–68.72% inhibition after 2 or 4 h post the final dose in the low group. And SCR‐7952 sustained in tumors rather than in plasma. The in vivo study demonstrated the inhibition effects of SCR‐7952 on tumor growth, as well as SAM synthesis.

It was noted that AG‐270 exhibited an inhibition effect on UGT1A1 with an IC_50_ of 1.1 µM, which could lead to elevation of bilirubin. However, SCR‐7952 exhibited low influence on metabolic enzyme UGT1A1, with IC_50_ > 100 µM (Figure 5C). In vivo study also found that SCR‐7952 exhibited no significant effect on mice plasma levels of bilirubin at a therapeutic dose (1.0 mg/kg) after 14 days oral treatment (Figure 5D). However, at the dose with comparable antitumor effects, AG‐270 (200 mg/kg) remarkably increased the plasma total bilirubin, direct bilirubin, and unconjugated bilirubin (Figure 5D).

### 2.5 The synergistic activity of SCR‐7952 in combination with different types of PRMT5 inhibitors

**FIGURE 6:**
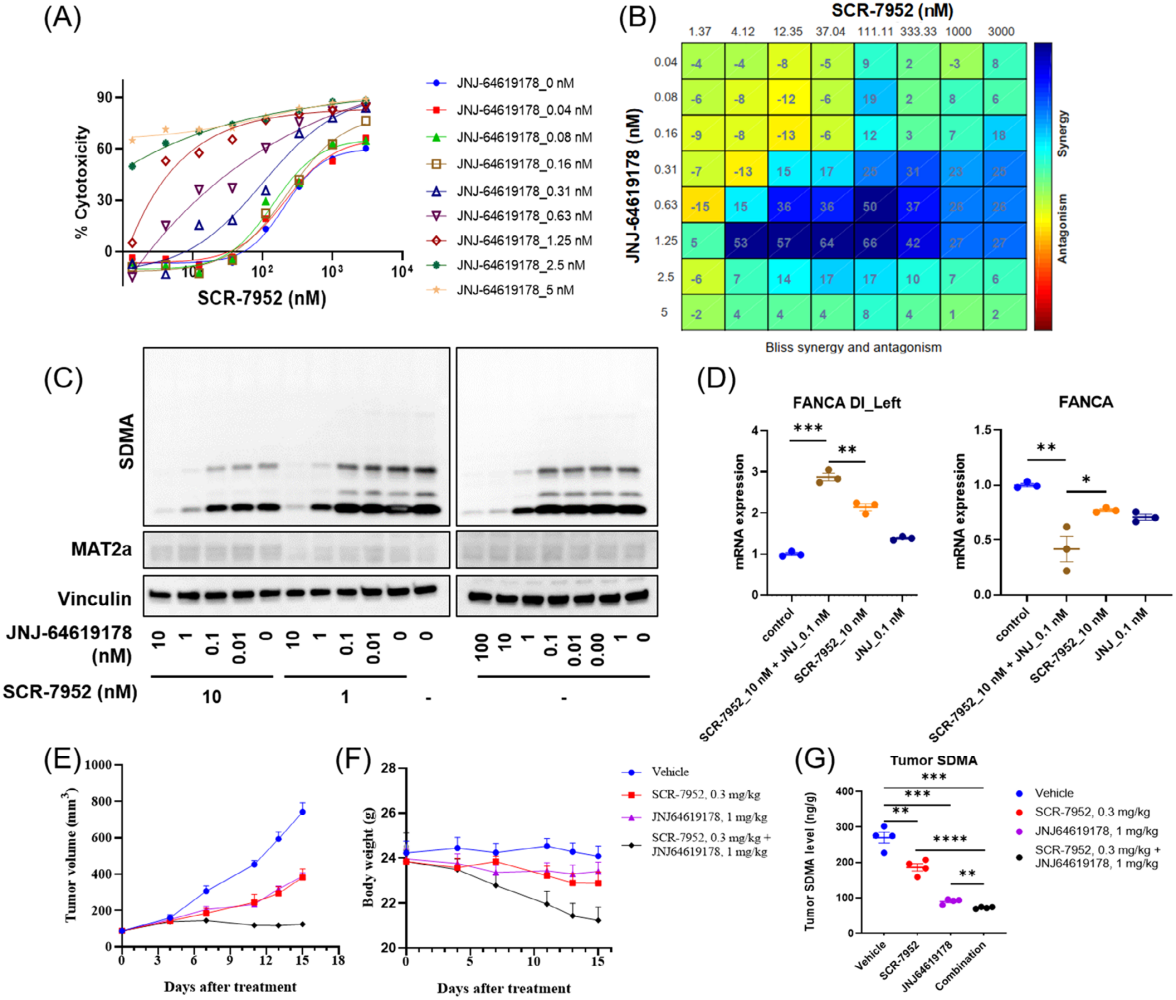
Synergistic activity of SCR‐7952 in combination with the PRMT5 inhibitor. (A) Drug dose–response curves for proliferation inhibition and (B) Bliss plots showing synergistic effects of the combination of SCR‐7952 and JNJ‐64619178 on HCT116 *MTAP* ^−/−^ cells for 8 days. (C) Immunoblot analysis of SDMA and MAT2A after combination treatment for 96 h, and (D) RT‐PCR of expressions of FANCA and the DI‐containing transcripts after SCR‐7952 treatment for 72 h. (E) Impact of SCR‐7952 and JNJ‐64619178 alone and in combination on tumor growth, (F) body weights, and (G) tumor SDMA levels on HCT116 *MTAP* ^−/−^ tumor‐bearing mice (mean ± SEM, *n* = 8).

PRMT5, one of the downstream of MAT2A, represents a synthetic lethal target in *MTAP*‐deficient cells.^23^ The inhibition of MAT2A leads to a reduction of PRMT5 methyltransferase activity, which catalyzes the formation of SDMAs from MAT2A product SAM. To explore better antitumor activities for *MTAP*‐deleted tumors, a combination of SCR‐7952 and different types of PRMT5 inhibitors were conducted on HCT116 *MTAP* ^−/−^ cells across a range of concentrations, including SAM‐competitive inhibitor JNJ‐64619178, MTA‐cooperative inhibitor MRTX‐1719, and substrate‐competitive PRMT5 inhibitor GSK3326595. The synergistic interactions of SCR‐7952 with JNJ‐64619178 or MRTX‐1719 on cell growth were elicited (Figures 6A and S4C). The Bliss model was used to calculate synergy scores, and strong synergistic effects were observed when Bliss scores were up to 20 (Figures 6B and S4D). The synergistic effect was also observed in the *MTAP*‐deleted (*CDKN2A*‐deleted) pancreatic cancer cell line KP4 (Figure S4A,B). However, the synergistic effect between SCR‐7952 and the substrate‐competitive PRMT5 inhibitor GSK3326595 was much weaker (Figure S4E,F).

Considering the impairment of MAT2A inhibition on PRMT5 activity, the combination of SCR‐7952 and JNJ‐64619178 may aggravate the inhibition of PRMT5 and its downstream. Thus, the SDMA marks and DI transcripts were assessed (Figure 6C,D). Similar to the MAT2A inhibitor SCR‐7952, JNJ‐64619178 treatment also caused a reduction of SDMA and the increase of FANCA DI‐containing transcripts. The combination of SCR‐7952 and JNJ‐64619178 led to enhanced inhibition of SDMA levels, as well as significantly higher expression of FANCA DI‐containing transcripts and lower levels of FANCA mRNA than those of SCR‐7952 or JNJ‐64619178 alone.

Encouraged by the in vitro synergy effects between SCR‐7952 and JNJ‐64619178, an in vivo combination treatment study was conducted in the HCT116 *MTAP* ^−/−^ xenograft mouse model. While modest efficacy was observed upon treatment with either SCR‐7952 (0.3 mg/kg) or JNJ‐64619178 (1.0 mg/kg) alone, almost complete tumor stasis was achieved when both agents were combined, significantly better than monotherapies (Figure 6E, combo vs. SCR‐7952 vs. JNJ‐64619178, TGI 94.4 vs. 55.0 vs. 54.0%, *p *< 0.0001). The mice were tolerable in the treatment group, and no severe adverse or animal abnormalities were observed during the study (Figure 6F).

To evaluate the in vivo impairment of PRMT5 activity after the combination treatment, tumor levels of SDMA were assessed (Figure 6G). Treatment with SCR‐7952 (0.3 mg/kg) or JNJ‐64619178 (1.0 mg/kg) alone both led to a remarkable reduction of SDMA levels. In the combination group, a significant reduction of SDMA level than that in single drug‐treated groups was observed, indicating a synergistic effect between SCR‐7952 and JNJ‐64619178.

## 3 DISCUSSION

Deletion of *CDKN2A*/*MTAP*, one of the most prevalent oncogenic events across all malignancies, occurs frequently in many human tumors, including 53% of glioblastomas and 26% of pancreatic cancers.^24^, ^25^ MTAP is critically involved in the methionine salvage pathway through the metabolization of MTA. The deletion of *MTAP* could lead MTA accumulation to an intracellular concentration of approximately 100 µM, causing a collateral vulnerability in the arginine methyltransferase PRMT, since MTA is a natural inhibitor of PRMT.^26^ Several PRMT inhibitors have been discovered and investigated in clinical trials, especially PRMT5 inhibitors, as PRMT5 plays an essential role in cancer stem cell survival, mRNA splicing, and DNA repair processes.^19^, ^27^ Despite preliminary efficacy, treatment‐related adverse events were observed in most trials, especially hematologic toxicity, including thrombocytopenia, anemia, and neutropenia.^19^ Thus, the upstream metabolic enzyme MAT2A, which primarily catalyzes the fundamental PRMT substrate SAM, was extended. The reduction of SAM, along with the accumulation of MTA, contributes to reducing PRMT5 activity and leads to antiproliferative effects in *MTAP*‐deleted cancer cells and tumors. Discovering the synthetic lethal interaction between MAT2A and MTAP, it was proposed that MAT2A inhibitors could be a promising strategy to suppress the proliferation of *MTAP*‐deleted cancers.^12^ However, to date, there have been only a few researches on this therapeutic approach.

Early studies have found the MAT2A inhibition effect of cycloleucine, a methionine analog.^28^ But the low affinity and the amino acid mimetic structure of cycloleucine limited its application. Recently, a potent allosteric MAT2A inhibitor PF‐9366 has been demonstrated to efficiently block SAM synthesis. However, the antiproliferation effects were inadequate in Huh‐7 cells and *mixed‐lineage leukemia*‐rearranged (*MLL*r) leukemia, with IC_50_ of about 10 µM.^17^, ^29^ Immunoblot analysis showed an upregulation of MAT2A expression after PF‐9366 treatment, which could probably act as a feedback mechanism. A series of MAT2A inhibitors have been discovered with potent effects using the structure‐based drug discovery, but their applications could be possibly limited by low solubility and poor pharmacokinetic properties.^13^, ^15^, ^16^ The most potent inhibitor among these compounds exhibited an IC_50_ of 22 nM on MAT2A in the biochemical assay and 250 nM on antiproliferation in HCT116 *MTAP*‐knockout cells. But the in vivo dosage reached to 50 mg/kg in the HCT116 *MTAP*‐knockout xenograft mouse model to exert an antitumor effect. Only AG‐270 and IDE397 were reported to undergo clinical trials in patients suffering from lymphoma or solid tumors (NCT03435250, NCT04794699, clinicaltrials.gov).^3^, ^14^ It was reported that IDE397 exhibited inhibition of MAT2A activity with IC_50_ of about 10 nM and antiproliferation effect on *MTAP* ^−/−^ cancer cells with IC_50_ of about 20 nM.^30^ But the in vivo dosage also reached to 10 mg/kg in a non‐small cell lung cancer PDX model.

In this study, we characterized a potent and allosteric MAT2A inhibitor SCR‐7952, exhibiting high affinity and high specificity using SPR and biochemical assays. Kinetic analysis and co‐EM assay indicated the allosteric binding site of SCR‐7952 on MAT2A. The cryo‐EM structure shows that SCR‐7952 binds at different binding sites from the substrate SAM. The binding of SCR‐7952 traps the reaction product by the closure of the α helix gate in the MAT2A active site. From the alignment of SCR‐7952 and MAT2B, it was found that SCR‐7952 binds at the same allosteric pocket as MAT2B does. Mat2B behaved as a negative regulator of MAT2A in the high levels of methionine or SAM. Similar binding sites were observed from previously reported MAT2A inhibitors AG‐270 and PF‐9366. PF‐9366 exhibited similar behavior with MAT2B, increasing the affinity of Mat2A for its substrate methionine, decreasing maximum velocity, and shifting Mat2A sensitivity to product inhibition.^17^ Also, the inhibitory activity of PF‐9366 reduced with the increase of MAT2B, indicating that these inhibitors probably competed with MAT2B for binding to a shared allosteric site of MAT2A.

The structure of SCR‐7952 is surrounded by a hydrophobic protein environment composed of aliphatic portions. Similar to AG‐270, SCR‐7952 maintains two hydrogen bond interactions with Arg313, which is necessary for small molecular binding to MAT2A. SCR‐7952 also maintains CH‐Pi interactions with Phe333, different from AG‐270, which maintains Pi–Pi interactions with Phe18. Compared with AG‐270, SCR‐7952 possesses a cyclopropyl structure, which contains good hydrophobic interactions with Tyr335 and Trp274. The alignment of SCR‐7952 and AG‐270 also shows that three parallel rings of SCR‐7952 possess more stable conformation and interactions with MAT2A. Besides, the molecular of SCR‐7952 was relatively small, while the binding and efficacy of SCR‐7952 remained fairly high. These interactions are reflected in the increased enzyme activity of SCR‐7952.

The inhibition of SCR‐7952 on MAT2A was demonstrated more potent than AG‐270 both in biochemical assay and in cells, though the expression of MAT2A protein was also upregulated in vitro after SCR‐7952 treatment. Previous studies have reported the upregulation of MAT2A expression after treatment of AG‐270 or PF‐9366, which may affect the antiproliferation activities of MAT2A inhibitors.^14^, ^17^ Despite the cellular adaptation, SCR‐7952 exhibited promising antiproliferative activity on *MTAP*‐deleted cancer cells, more potent and selective than AG‐270, which was presumably a result of its high enzyme activity. In vivo studies confirmed that SCR‐7952 presented a significantly better antitumor efficacy (TGI 82.9% at 3.0 mg/kg) than AG‐270 (TGI 52.0% at 200 mg/kg) in HCT116 *MTAP* ^−/−^ xenograft mouse model. Other MAT2A inhibitors, such as AZ9567 and Compound 28 exhibited similar in vitro potency and selectivity.^15^, ^16^ But SCR‐7952 showed in vivo antitumor effects at significantly lower therapeutic dosages than the reported MAT2A inhibitors, which was possibly due to good pharmacokinetic profiles. It was demonstrated by pharmacokinetic studies of SCR‐7952 (1.0 mg/kg) and AG‐270 (200 mg/kg) after multiple administration in xenograft mice (Table S4). The calculated AUC_0–24h_ free drug of SCR‐7952 and AG‐270 were 1442 and 3829 h × ng/mL. Considering the 8.7‐fold difference of the antiproliferative effect of SCR‐7952 and AG‐270 on HCT116 *MTAP* ^−/−^ cancer cells (34.4 vs. 300.4 nM), the significantly lower therapeutic dosage of SCR‐7952 was reasonable.

SDMA, a marker of PRMT5 activity, was significantly reduced both in vitro and in vivo upon treatment of SCR‐7952, corresponding to the reduction of SAM levels. MAT2A inhibition drives the downregulation of PRMT5 activity in cells, resulting in dysregulation of splicing and severe perturbations.^12^ Among the massive pre‐mRNA splicing events, the downregulation of FA DNA repair pathway attracted our attention. FA pathway coordinates a complex mechanism and employs unique nuclear protein complexes to the area of DNA damage, contributing to the resolution of R loops and the formation of DNA repair structures.^31^ FANCA, a component of the FA core complex, participates in the activation of DNA inter‐strand crosslink repair, as well as catalyzing single‐strand annealing and strand exchange.^32^ Here, we evaluated the expression of FANCA and its DI‐containing transcripts. SCR‐7952 treatment led to significant upregulation of FANCA DI‐containing transcripts, and in turn, reduction of total levels of FANCA. The partial loss of FA activity via DI‐mediated downregulation upon MAT2A inhibition results in exacerbated DNA damage, thus causing mitotic defects downstream.^12^ Taken together, the MAT2A inhibitor SCR‐7952 drives the downregulation of PRMT5 activity, causing dysregulation of splicing and partial FA pathway deficiency. These effects contribute together to the antiproliferation of *MTAP*‐deficient cancer cells.

Though with potent antitumor effects, the feedback loop of upregulation of MAT2A expression may still limit the application of MAT2A inhibitors. Clinical researches evaluated the antitumor activity of a MAT2A inhibitor IDE397 in patients with *MTAP*‐deleted squamous NSCLC. The preliminary data showed 75% molecular responses (3/4, up to 50% circulating tumor DNA decrease), but only one PR was observed (NCT04794699, clinical trials.gov). The result indicated that the effect of the MAT2A inhibitor as a single agent was not strong enough, and thus combination strategies were needed. Considering the same mechanism and downstream of MAT2A and PRMT5, it was speculated that there could be synergistic effects between MAT2A inhibitors and PRMT5 inhibitors. Several types of PRMT5 inhibitors have been discovered and investigated in preclinical and clinical studies, but only a few publications reported the combination of MAT2A and PRMT5 inhibitors. One study evaluated the selective MAT2A inhibitor IDE397 in combination with MTA‐cooperative PRMT5 inhibitors in *MTAP*‐deleted lung adenocarcinoma and pancreas cancer models H838 and BXPC3.^21^, ^30^ Another study reported a combination of an MAT2A inhibitor GH31 with a MTA‐cooperative PRMT5 inhibitor GH56.^20^ However, the mechanism of the synergistic effects was not demonstrated. Also, the combination of MAT2A inhibitors with different types of PRMT5 inhibitors has not been systematically studied.

In this study, a systematic evaluation of different PRMT5 inhibitors was explored, including SAM‐competitive (JNJ‐64619178), substrate‐competitive (GSK3326595), and MTA‐cooperative (MRTX‐1719) ones. It was an innovative discovery that the combination of SCR‐7952 with SAM‐competitive or MTA‐cooperative PRMT5 inhibitors could sensitize the antiproliferative effects of each other, thus providing a larger therapeutic window for *MTAP*‐deleted tumors. The possible mechanism of combination with SAM‐competitive inhibitors is due to the reduction of SAM and thus aggravated inhibition of PRMT5 in tumors after MAT2A inhibition. The reduction of SAM could possibly lead to a remarkable sensitization of SAM‐competitive PRMT5 inhibitors that occupied the SAM‐binding pocket in PRMT5 to block substrate methylation.^33^ As for MTA‐cooperative PRMT5 inhibitors, their activities resulted from the binding and stabilization of the PRMT5•MTA complex. MTA competed with SAM for binding to PRMT5 as an endogenous inhibitor, and the reduction of SAM was deduced to sensitize the effect of MTA‐cooperative inhibitors in *MTAP*‐deleted cancer cells.^34^ However, the synergistic effect of SCR‐7952 with the substrate‐competitive PRMT5 inhibitor was not as promising. Substrate‐competitive inhibitors bound to the catalytic region of PRMT5, a site distinct from MTA and SAM, and prevented its catalytic effects.^35^ By the way, because PRMT5 is required for the viability of all cells, and hematopoietic cells in particular, the SAM‐competitive inhibitors exhibited hematologic toxicities and did not appear to have a sufficiently wide therapeutic index.^36^ But MTA‐cooperative inhibitors exhibited minimal changes in hematopoietic cells. So, the combination of SCR‐7952 with MTA‐cooperative ones was recommended in further clinical studies.

Metabolic enzymes are important for the degradation of endogenous metabolites, drugs, and other toxicants. SCR‐7952 exhibited little influence on metabolic enzymes. Different from AG‐270, SCR‐7952 exhibited low inhibition effect on UGT1A1, with IC_50_ > 100 µM (AG‐270, IC_50_ of 1.1 µM). UGT1A1‐mediated glucuronidation is an indispensable step in the metabolism and excretion of endogenous bilirubin, as well as many drugs including irinotecan and losartan.^37^ Inhibition of UGT1A1 leads to hyperbilirubinemia, which may cause damage to the nerve and liver.^18^ Also, there could be drug‐drug interactions with AG‐270 due to the inhibition of UGT1A1, thus limiting the simultaneous administration with UGT1A1‐metabolized drugs. So, SCR‐7952 possesses advantages in this respect.

Our data demonstrated antitumor effect of MAT2A inhibitor SCR‐7952 and the synergetic effect with PRMT5 inhibitors on *MTAP*‐deleted tumors, via the inhibition of PRMT5 activity and splicing perturbations. Although interesting discoveries were revealed in our studies, there were also some limitations. First, we demonstrated that SCR‐7952 regulated PRMT5 activity and specific splicing perturbations, according to reported literatures. But there might be further and additional mechanistic insights, which would be revealed by using high‐throughput technologies such as RNA sequencing and metabolomics in our later studies. Second, we demonstrated the synergetic effects using only one representative inhibitor of each type of PRMT5 inhibitors. Further studies would evaluate other MAT2A inhibitors with more PRMT5 inhibitors in combination study, to validate the difference of synergetic effects among different types of PRMT5 inhibitors. Further researches were also needed to verify the speculation of the mechanism that SCR‐7952 exhibited synergetic effects with SAM‐competitive or MTA‐cooperative PRMT5 inhibitors, but not substrate‐competitive ones. Also, there were several clinical trials investigating the combination effects of MAT2A inhibitors AG‐270, S095033, and IDE397 with other anticancer agents including taxanes and sacituzumab govitecan (ClinicalTrials.gov NCT03435250, NCT05312372, NCT04794699), indicating that more possible combination strategies of SCR‐7952 with other anticancer agents need further exploration.

## 4 CONCLUSION

We discovered a potent and specific allosteric MAT2A inhibitor SCR‐7952, with promising inhibition on the growth of *MTAP*‐deleted cancers, via the regulation of PRMT5 activity and its downstream. Furthermore, the combination of SCR‐7952 with SAM‐competitive or MTA‐cooperative PRMT5 inhibitors supported further investigation and potential development of an effective strategy for larger therapeutic windows on *MTAP*‐deleted cancers. Considering the clinical safety, it was suggested to combine SCR‐7952 with MTA‐cooperative PRMT5 inhibitors.

## 5 MATERIALS AND METHODS

### 5.1 Reagents

SCR‐7952 and AG‐270 were synthesized according to patents WO2022143864 and WO2018039972. JNJ‐64619178 (HY‐101564), MRTX‐1719 (HY‐139611), and GSK3326595 (HY‐101563) were purchased from MCE. MAT2A protein (Cat. 210405‐HWR‐20210429) was bought from Pharmaron. SAM (Cat. A4377), l‐methionine (Cat. M9625), and ATP (Cat. A7699) were bought from Sigma. SAM‐*d*_3_ (Cat. A291532) was bought from Toronto Research Chemicals. Rabbit anti‐SDMA (Cat. 13222S), rabbit anti‐MTAP (Cat. 62765), and rabbit anti‐HSP90 (Cat. 4877) were bought from CST. Rabbit anti‐MAT2A (Cat. ab186129) and rabbit anti‐vinculin (Cat. ab219649) were bought from Abcam. Phosphate Assay Kit‐PiColorLock™ was bought from Abcam. CellTiter‐Glo 2.0 Cell Viability Assay Kit was bought from Promega. RNeasy Plus Mini Kit was bought from QIAGEN. PrimeScript™ II 1st Strand cDNA Synthesis Kit and TB Green® Fast qPCR Mix Kit were bought from TAKARA. The primer pairs were in Table S1.

### 5.2 Cell lines

HCT116 colon carcinoma parental (WT) cells with *MTAP* ^−/−^ isogenic clone were established by KYinno Biotechnology Co., Ltd. (Beijing, China). A549, MIA PaCa‐2, and WI‐38 cell lines were obtained from ATCC. NCI‐H838 was obtained from Cobioer Biosciences. HCT116 WT and KO *MTAP* cells were cultured in McCoy's 5A media containing 10% FBS. A549 cells were cultured in DMEM media containing 10% FBS. MIA PaCa‐2 cells were cultured in DMEM media containing 10% FBS and 2.5% horse serum. NCI‐H838 cells were cultured in PRMI 1640 media containing 10% FBS. WI‐38 cells were cultured in EMEM media containing 10% FBS.

### 5.3 Mice and ethics statements

Nu/Nu female mice (6–12 weeks old; Beijing Vital River Laboratory Animal Technology Co., Ltd.) were used for in vivo xenograft studies. All experimental procedures involving animals and their care were in accordance with the State Council Regulations for Laboratory Animal Management (Enacted in 1988) and approved by the Institutional Animal Care and Use Committee of Jiangsu Simcere Pharmaceutical Co., Ltd. (IACUC; Approval No. AP‐056).

### 5.4 In vitro enzymology

For determination of the inhibitory potency of compounds against the MAT2A, the protein was diluted to a final dose of 4 µg/mL in assay buffer (50 mM Tris, 50 mM KCl, 15 mM MgCl_2_, 0.3 mM EDTA, 0.005% [w/v] bovine serum albumin [BSA]). The test compound was prepared in dimethyl sulfoxide (DMSO). An 80 nL volume of compound dilution was added to the 384‐well plate using an Echo 650 (Labcyte, USA). Then, 40 µL of enzyme dilution was added and the mixture was incubated for 120 min at room temperature. The enzymatic assay was initiated by adding 40 µL of substrate mix (a final dose of 400 µM ATP and a final dose of 200 µM l‐methionine in assay buffer), and the mixture was incubated for a further 90 min at room temperature. The liberated phosphate released by the enzyme in stoichiometric amounts by the production of SAM was measured using the Phosphate Assay Kit—PiColorLock™ (Abcam, UK). Velocity curves were fit to the standard Michaelis‐Menten equation to yield *V*_max_ and *K*_m_.

### 5.5 SPR analysis

SPR experiments were performed using a BIAcore 8K instrument (Cytiva) at 25°C. A series S sensor chip SA (Cytiva) was docked into the system in 20 mM Bicine‐Na, pH 7.5, 100 mM KCl, 5 mM MgCl_2_, 0.1 mM TCEP, 0.05% (w/w) Tween‐20, and 2% (v/v) DMSO. The MAT2A protein was captured by Penta‐anti‐His antibody, and the affinity of SCR‐7952 was tested over a suitable dose–response range (12.5–200 nM) by single‐cycle kinetics. The kinetic parameters of SCR‐7952 on MAT2A were fitted via a 1:1 binding model.

### 5.6 cryo‐EM of SCR‐7952 with MAT2A protein

The homogeneity of the sample was first examined by negative‐stain cryo‐EM with 0.7% (w/v) uranyl acetate. To prepare grids for cryo‐EM, the freshly purified MATA2 protein was incubated with SCR‐7952 and SAM at a molar ratio of 1:3:3 for 1 h and centrifuged at 14,000×*g* for 5 min to remove potential protein aggregates. The protein concentration was measured with a NanoDrop spectrophotometer (Thermo Fisher Scientific) and adjusted to 5 mg/mL. A total of 4 µL aliquot was applied to glow‐discharged grids (300 mesh R0.6/1 Au grids; Quantifoil) and frozen with a Vitrobot Mark IV (Thermo Fisher Scientific) set at 4°C and 100% humidity. The grids were blotted for 2 s with a blot force of −2 and then plunged into liquid nitrogen‐cooled ethane. Data collection was performed on a 300 kV Titan Krios electron microscope (Thermo Fisher Scientific) at a nominal magnification of ×81,000, corresponding to a calibrated pixel size of 1.055 Å at the specimen level. Images were collected using a defocus range of −1.2 to −2.2 µm in super‐resolution counting mode.

The movie stacks were motion‐corrected and dose‐weighted in MotionCor2^38^. The contrast transfer function (CTF) parameters were determined with CTFFIND4 implemented in RELION‐3.^39^, ^40^ A total of 679,996 particles were automatically picked from 1,000 micrographs with Gautomatch (https://www.mrc‐lmb.cam.ac.uk/kzhang/Gautomatch/) using the selected 2D averages from a small subset of the data as templates. The particles were extracted into 200 × 200 boxes. After 2D classification, 158,968 particles with clear secondary structures were selected and subjected to ab initials reconstruction with Cryosparc.^41^ 125,837 particles were selected and the alignment parameters are optimized in CryoSPARC nonuniform refinement to generate a map at 3.16 Å with D2 symmetry.

We used the predicted structure of human MATA2 from AlphaFold as the starting model and fitted it into the density map with Chimera.^42^ All manual model building was performed with Coot.^43^ The atomic model was refined by using phenix.real_space_refine.^44^

### 5.7 Intracellular SAM inhibition assay

MAT2A inhibition in the cellular environment was monitored by measurement of the abundance of its product SAM in HCT116 *MTAP* ^−/−^ cells. HCT116 *MTAP* ^−/−^ cells were plated in 96‐well cell culture plates at 50,000 cells per well and allowed to attach overnight at 37°C in 5% CO_2_. Compounds were added in a dose–response format to generate a 10‐point dose–response curve. Doses were started at a 10 µM top concentration with 1:5 serial dilution. After 6 h of incubation, the medium was removed and cells were extracted with 50% methanol containing 1 M acetic acid for SAM metabolite measurement by UPLC‐MS/MS.

The standard SAM solution was prepared in a surrogate matrix of 50% methanol containing 1 M acetic acid with a concentration range of 3–1000 ng/mL. The quality control sample was prepared in the same surrogate matrix. SAM‐*d*_3_ was used as the internal standard (IS). A Waters UPLC system coupled with a SCIEX Triple Quad™ 6500^+^ mass spectrometer (Waters, USA) was used to analyze the samples. Samples were separated on an ACQUITY UPLC^®^ BEH amide column (2.1 × 50 mm, 1.7 µm). The mobile phase consisted of 5% acetonitrile containing 5 mM NH_4_OAc and 1% (v/v) formic acid (A) and 95% acetonitrile containing 5 mM NH_4_OAc and 1% (v/v) formic acid (B). A linear gradient elution program for the analysis was as follows: 0–0.3 min, 20% A; 0.3–0.8 min, 20–47% A; 0.8–1.5 min, 47% A. The flow rate was 0.5 mL/min. The column temperature was 60°C. An aliquot of 3 µL was injected for analysis. The optimized mass spectrometry parameters in the positive ion mode were as follows: nebulizer gas, 55 psi; auxiliary heater gas, 55 psi; curtain gas, 35 psi; collision gas, 8 psi; spray voltage, 5500 V; probe heater temperature: 450°C. SAM and SAM‐*d*_3_ were detected with multiple‐reaction monitoring of a mass transition pair at *m*/*z* 399.2/250.2 and 402.2/301.2, respectively.

### 5.8 Proliferation assay

Cells were plated in 96‐well cell culture plates and allowed to attach overnight at 37°C in 5% CO_2_. Compounds diluted in DMSO were added to cells in a dose–response format for 6 days. The final concentration of DMSO was 0.1% in media. Cell proliferation was measured using CellTiter‐Glo Cell Viability Assay (Promega). The IC_50_ values were calculated using the GraphPad Prism 9. Drug combination effects were analyzed using Combenefit software.^45^

### 5.9 Xenograft studies

Subcutaneous injections of HCT116 *MTAP* ^−/−^ cells were performed (5 × 10^6^ cells in 100 µL) into Nu/Nu female mice. Mice were observed daily for the development of tumors. Six days after tumor implantation, mice were randomly distributed into different groups. SCR‐7952, AG‐270, JNJ‐64619178, or vehicle (water with 5% *N*‐methylpyrrolidone and 10% solutol HS‐15) were daily administrated by oral gavage. Tumor volumes and body weights were recorded three times per week. Tumor volumes were measured in two dimensions using a caliper, and the volume was expressed in mm^3^ using the formula: *V* = (*L* × *W* × *W*)/2, where *V* is tumor volume, *L* is tumor length (the longest tumor dimension), and *W* is tumor width (perpendicular to *L*). The tumor growth inhibition rate (TGI%) of each dosing group was calculated according to the following formula: %TGI = (1 − (*V*_t(treatment group)_ − *V*_0(treatment group)_)/(*V*_t(vehicle group)_ − *V*_0(vehicle group)_)) × 100%, where *V*_t_ is average tumor volume of a dosing group on a specific day, *V*_0_ is average tumor volume of a dosing group on the initial day. At the end of the study, plasma and tumor tissues were collected and stored at −80°C for further measurement.

### 5.10 In vivo SAM measurement by LC–MS/MS

The concentration of SAM in plasma and tissue samples was determined using a UPLC–MS/MS method. For plasma samples, an aliquot of 20 µL plasma sample was added with 400 µL extraction buffer (1 M acetic acid in MeOH), then vortexed and centrifuged at 16,000 *g* for 5 min. The supernatant was treated as the homogenized tissue sample, and the sample dilution factor was 21. For tumor samples, the tumor tissues were homogenized with 9 volumes (*v*/*w*) of extraction buffer, then vortexed and centrifuged at 16,000 *g* for 5 min. The supernatant was treated as the homogenized tissue sample. An aliquot of 30 µL tissue sample was added with 600 µL extraction buffer, then vortexed and centrifuged at 16,000 *g* for 5 min. The total sample dilution factor is 210. An aliquot of 60 µL diluted samples, standards, or QCs was added with 400 µL extraction buffer with IS (SAM‐*d*_3_, 50 ng/mL in extraction buffer). The mixture was vortexed at 250 *g* for 10 min and centrifuged at 16,000 *g* for 5 min. An aliquot of 4 µL supernatant was injected for UPLC–MS/MS analysis. The standard calibration curve was in a 1.0–300 ng/mL concentration range. UPLC–MS/MS method for SAM detection was performed as mentioned before.

### 5.11 Statistical analysis

Data were presented as mean ± SEM, with the indicated sample size (*n*) representing biological replicates. Group allocation was performed in a random manner. Statistical significance was determined by two–way analysis of variance (ANOVA). *p* < 0.05 was considered statistically significant.

## AUTHOR CONTRIBUTIONS

All authors read and approved the final version of the manuscript. *Zhiyong Yu, Yi Kuang, Liting Xue, and Xuan Ma*: contributed equally to conceptualization, methodology, analysis, and writing‐original draft for this work. *Grace Xue*: contributed to methodology and data curation. *Tingting Li, Linlin Yuan, Mengying Li, Zhen Li, Feng Tang, Jianxing Tang, and Jinwen Shan*: contributed to investigation, methodology, data curation, formal analysis, and visualization. *Weijie Wang and Renhong Tang*: contributed to conceptualization, validation, supervision, resources, and funding acquisition. *Feng Zhou*: contributed to conceptualization, validation, supervision, investigation, and writing—review and editing.

## CONFLICT OF INTEREST STATEMENT

Z. Y., Y. K., L. X., T. L., L. Y., M. L., Z. L., F. T., J. T., J. S., R. T., and F. Z. were employees of Simcere Zaiming Pharmaceutical Co., Ltd. and Jiangsu Simcere Pharmaceutical Co., Ltd. at the time of the study. Simcere Zaiming Pharmaceutical Co., Ltd. provided financial support to the current study. Other authors declare no conflict of financial interest.

## ETHICS STATEMENT

All experimental procedures involving animals and their care were in accordance with the State Council Regulations for Laboratory Animal Management (Enacted in 1988) and approved by the Institutional Animal Care and Use Committee of Jiangsu Simcere Pharmaceutical Co., Ltd. (IACUC; Approval No. AP‐056).

## Supporting information

Supporting Information

## Data Availability

All data generated or analyzed in this study were included in this published article.
