# Supplementary material for: SCR‐7952, a highly selective MAT2A inhibitor, demonstrates synergistic antitumor activities in combination with the S‐adenosylmethionine‐competitive or the methylthioadenosine‐cooperative protein arginine methyltransferase 5 inhibitors in methylthioadenosine phosphorylase‐deleted tumors
Source: MedComm (2020). 2024 Sep 20;5(10):e705. doi: 10.1002/mco2.705 (PMC11413503; doi:10.1002/mco2.705)
Supplement: Supplementary file 1 — Supporting Information [file MCO2-5-e705-s001.docx]

**Supplementary Information**

**SCR-7952, a highly selective MAT2A inhibitor demonstrates synergistic anti-tumor activities in combination with the *S*-adenosylmethionine-competitive or the methylthioadenosine-cooperative protein arginine methyltransferase 5 inhibitors in methylthioadenosine phosphorylase-deleted tumors**

**Zhiyong Yu^1, 3, #^, Yi Kuang^3, #^, Liting Xue^1, 3, #^, Xuan Ma^2, #^, Tingting Li^3^ , Linlin Yuan^1^, Mengying Li^3^, Grace Xue^4^, Zhen Li^3^, Feng Tang^1^, Jianxing Tang^3^, Jinwen Shan^3^, Weijie Wang^2, *^, Renhong Tang^1, 3, *^, and Feng Zhou^1, 3, *^**

**1** **State Key Laboratory of Neurology and Oncology Drug Development, Nanjing, China.**

**2 Department of Thoracic Surgery, The Affiliated Xiangshan Hospital of Wenzhou Medical University, Wenzhou, Zhejiang, China.**

**3** **Simcere Zaiming Pharmaceutical Co., Ltd., Shanghai, China.**

**4 Weston High School, 444 Wellesley St, Weston, MA 02493, USA.**

**# These authors contributed equally to this work.**

*** Corresponding Authors, who jointly supervised this work:**

**Renhong Tang:** renhong.tang@simceregroup.com

**Weijie Wang:** wangweijie19830720@126.com

**Feng Zhou:** zhoufeng2@simcere.com**,** fengz504@icloud.com

**Figure legends**

**Figure S1.** Model of Mat2A in the absence of substrate and inhibitors.

(a) The structures of MAT2A SAM free form, in the presence of SAM, in the presence of SAM and MAT2B, and in the presence of SAM and SCR-7952. (b) The alignment of MAT2A·SAM·SCR-7952 and MAT2A·SAM·MAT2B (PDB code: 4NDN). (c) The binding pose and schematic diagram of AG-270 (PDB code: 7KCC; pink stick model).

**Figure S2.** The anti-proliferative effects of SCR-7952 (a) and MTAP expression (b) in cell lines.

**Figure S3.** Reduction of SAM in HCT116 *MTAP*^-/-^ xenografted mice.

(a) Tumor and (b) plasma SAM levels 2 h or 4 h after the final dose in HCT116 *MTAP*^-/-^ xenografted mice treated with SCR-7952 or vehicle for 16 days. P values were calculated using Two-Way ANOVA. ***p* <0.01, ****p* < 0.001.

**Figure S4.** Synergistic activity of SCR-7952 in combination with PRMT5 inhibitors on KP4 or HCT116 *MTAP*^-/-^ cells.

Drug dose-response curves for proliferation inhibition and Bliss plots showing synergistic effects of combination of SCR-7952 (a) with JNJ-64619178 on KP4 cells, (b) with MRTX-1719 on HCT116 *MTAP*^-/-^ cells, and (c) with GSK3326595 on HCT116 *MTAP*^-/-^ cells for 8 days.

**Table legends**

**Table S1.** Primer pairs used in this study.

**Table S2.** Inhibition effects of SCR-7952 and AG-270 on MAT2A and cell proliferation.

**Table S3.** Mini-profiling panel examining off-target inhibition of SCR-7952.

**Table S4.** Plasma concentration of SCR-7952 and AG-270 after treatment in HCT116 *MTAP*^-/-^ xenograft mice.

**Figure S1. Model of Mat2A in the absence of substrate and inhibitors.**

(a) The structures of MAT2A SAM free form, in the presence of SAM, in the presence of SAM and MAT2B, and in the presence of SAM and SCR-7952. (b) The alignment of MAT2A·SAM·SCR-7952 and MAT2A·SAM·MAT2B (PDB code: 4NDN). (c) The binding pose and schematic diagram of AG-270 (PDB code: 7KCC; pink stick model).

**Figure S2. The anti-proliferative effects of SCR-7952 (a) and MTAP expression (b) in cell lines.**

**Figure S3. Reduction of SAM in HCT116 *MTAP*^-/-^ xenografted mice.**

(a) Tumor and (b) plasma SAM levels 2 h or 4 h after the final dose in HCT116 *MTAP*^-/-^ xenografted mice treated with SCR-7952 or vehicle for 16 days (mean ± SEM, n = 4). P values were calculated using unpaired Student’s t test. ***p* <0.01, ****p* < 0.001.

**Figure S4. Synergistic activity of SCR-7952 in combination with PRMT5 inhibitors on KP4 or HCT116 *MTAP*^-/-^ cells.**

Drug dose-response curves for proliferation inhibition and Bliss plots showing synergistic effects of combination of SCR-7952 (a) with JNJ-64619178 on KP4 cells, (b) with MRTX-1719 on HCT116 *MTAP*^-/-^ cells, and (c) with GSK3326595 on HCT116 *MTAP*^-/-^ cells for 8 days.

**Table S1. Primer pairs used in this study.**

| **Target** |  | **Sequence** |
| --- | --- | --- |
| FANCL DI_Left | Forward | GTCACTGATCAAGCAAAAATGG |
|  | Reverse | CTTCTTGCATTCATGGCATT |
| FANCL DI_Right | Forward | ATGTGAAGCAGGGAGAGTCG |
|  | Reverse | TCCATAACATCCCAGAATGC |
| FANCL | Forward | GGTAGACCCCAGGCATCCTA |
|  | Reverse | CAGGATAGCACGAGCTGGAA |
| FANCA_DI_Left | Forward | TTGGCTGCTGGAGTACAGG |
|  | Reverse | AGACACCTCCCTGCTGCAC |
| FANCA_DI_Right | Forward | CAAGGCACCCTCCCTTTC |
|  | Reverse | TCTTCAGAGGATCTGTGGAAAT |
| FANCA | Forward | TCTGCTGCTGAAGAGAAGCC |
|  | Reverse | TCATGGAGGCTCTCAGCTCT |

**Table S2. IC_50_ values of SCR-7952 and AG-270 on MAT2A and cell proliferation.**

|  |  | **SCR-7952 (nM)** | **AG-270 (nM)** |
| --- | --- | --- | --- |
| MAT2A enzyme | | 18.7 | 68.3 |
| Cellular SAM levels | | 1.9 | 5.8 |
| Anti-proliferation | HCT116 *MTAP*^-/-^ | 34.4 | 300.4 |
|  | HCT116 WT | 487.7 | 1223.3 |
|  | NCI-H838 | 4.3 | 93.9 |
|  | MIA PaCa-2 | 19.7 | 935.4 |
|  | A549 | 123.1 | 646 |
|  | WI-38 | 4306.9 | 1413 |

**Table S3. Mini-profiling panel examining off-target inhibition of SCR-7952.**

| GPCR Targets | Agonism | Antagonism |  | Ion Channels | Opener | Blocker |
| --- | --- | --- | --- | --- | --- | --- |
|  | EC_50_ (nM) | IC_50_ (nM) |  |  | EC_50_ (nM) | IC_50_ (nM) |
| ADORA2A | >10,000 | >10,000 |  | CAV1.2 | - | >10,000 |
| ADRA1A | >10,000 | >10,000 |  | GABAA | >10,000 | >10,000 |
| AVPR1A | >10,000 | >10,000 |  | hERG | - | >10,000 |
| CCKAR | >10,000 | >10,000 |  | HTR3A | >10,000 | >10,000 |
| CHRM1 | >10,000 | >10,000 |  | KvLQT1/mink | >10,000 | >10,000 |
| CHRM3 | >10,000 | >10,000 |  | nAChR(a4/b2) | >10,000 | >10,000 |
| EDNRA | >10,000 | >10,000 |  | NAV1.5 | - | >10,000 |
| HRH1 | >10,000 | >10,000 |  | NMDAR (1A/2B) | >10,000 | >10,000 |
| HTR2A | >10,000 | >10,000 |  |  |  |  |
| HTR2B | >10,000 | >10,000 |  | Kinases | Inhibitor |  |
| ADRA2A | >10,000 | >10,000 |  |  | IC_50_ (nM) |  |
| ADRB1 | >10,000 | >10,000 |  | INSR | >10,000 |  |
| ADRB2 | >10,000 | >10,000 |  | LCK | >10,000 |  |
| CHRM2 | >10,000 | >10,000 |  | ROCK1 | >10,000 |  |
| CNR1 | >10,000 | >10,000 |  | VEGFR2 | >10,000 |  |
| CNR2 | >10,000 | >10,000 |  |  |  |  |
| DRD1 | >10,000 | >10,000 |  | NHR Targets | Agonism | Antagonism |
| DRD2S | >10,000 | >10,000 |  |  | EC_50_ (nM) | IC_50_ (nM) |
| HRH2 | >10,000 | >10,000 |  | AR | >10,000 | >10,000 |
| HTR1A | >10,000 | >10,000 |  | GR | >10,000 | >10,000 |
| HTR1B | >10,000 | >10,000 |  |  |  |  |
| OPRD1 | >10,000 | >10,000 |  | Non-Kinase Enzymes | Inhibitor |  |
| OPRK1 | >10,000 | >10,000 |  |  | IC_50_ (nM) |  |
| OPRM1 | >10,000 | >10,000 |  | AChE | >10,000 |  |
|  |  |  |  | COX1 | >10,000 |  |
| Transporters | Blocker |  |  | COX2 | >10,000 |  |
|  | IC_50_ (nM) |  |  | MAOA | >10,000 |  |
| DAT | >10,000 |  |  | PDE3A | >10,000 |  |
| NET | >10,000 |  |  | PDE4D2 | >10,000 |  |
| SERT | >10,000 |  |  |  |  |  |

**Table.S4. Plasma concentration of SCR-7952 and AG-270 after treatment in** **HCT116 *MTAP*^-/-^ xenograft mice.**

|  | SCR-7952 (1.0 mg/kg) | AG-270 (200 mg/kg) |
| --- | --- | --- |
| AUC_0-24h total_ (h×ng/mL) | 15844 | 382904 |
| AUC_0-24h_ _free drug_ (h×ng/mL) | 1442 | 3829 |
